# Diagnostic and Prognostic Value of Plasma lncRNA SRA1 in Chronic Heart Failure

**DOI:** 10.31083/j.rcm2505178

**Published:** 2024-05-20

**Authors:** Yiming Yu, Xiao Ge, Lifang Cao, Feng Li

**Affiliations:** ^1^Department of General Practice, The First Affiliated Hospital of Weifang Medical University, Weifang People's Hospital, 261041 Weifang, Shandong, China

**Keywords:** lncRNA SRA1, chronic heart failure, BNP, left atrial end-systolic diameter, left ventricular end-diastolic diameter

## Abstract

**Background:**

The pathogenesis and development of chronic heart failure (CHF) may involve long non-coding ribonucleic acid (lncRNA) steroid receptor RNA activator 1 (SRA1), a known cardiomyopathy risk factor and regulator of cardiac myofibroblast activation. This study aimed to investigate the application of SRA1 in the early detection and prediction of CHF.

**Methods:**

SRA1 plasma expression was determined in CHF patients and healthy individuals/using real time-quantitative polymerase chain reaction (RT-qPCR). The diagnostic and prognostic value of SRA1 was assessed using receiver operating curve (ROC) and Cox regression analyses.

**Results:**

Compared with the healthy controls, the patients with CHF had increased brain natriuretic peptide (BNP) levels, left atrial end-systolic diameter (LAD), left ventricular end-diastolic diameter (LVDd), and decreased left ventricular ejection fraction (LVEF). SRA1 was significantly upregulated in CHF patients as well as positively correlated with BNP level, LAD, and LVDd, and negatively correlated with LVEF. SRA1 could sensitively discriminate CHF patients from healthy individuals and was an independent predictor of adverse event-free survival in CHF patients.

**Conclusions:**

Upregulated plasma SRA1 can discriminate patients with CHF from healthy individuals and predict adverse outcomes in CHF patients. Thus, SRA1 is a potential molecular indicator for monitoring chronic heart failure development.

## 1. Introduction

Chronic heart failure (CHF) is a condition induced by abnormal heart structure and function, presenting with complex and nonspecific symptoms [1]. Moreover, the incidence and mortality rate of chronic heart failure are relatively high and have been progressively increasing posing a threat to the quality and safety of human life. It was reported that the rehospitalization rate of CHF patients was over 20%, and the mortality of hospitalized patients was about 10% in China [2, 3]. CHF is primarily diagnosed based on specific biomarkers, including brain natriuretic peptide (BNP) and NT-prosomal BNP (NT-proBNP) levels [4, 5]. Recently, identifying biomarkers that effectively screen CHF occurrence and monitor disease development has gained special attention. In addition to high sensitivity and specificity, an ideal biomarker should exhibit stability with a relatively long half-life in body fluid and be easily detectable.

Long non-coding RNAs (lncRNAs) carry extensive biological data and are involved in cellular function regulation. LncRNAs can be non-invasively detected in serum, plasma, and urine and have demonstrated outstanding clinical significance in cardiovascular diseases. For example, plasma lncRNA high expressed in hepatocellular carcinoma (HEIH) was found to be correlated with coronary artery disease prognosis [6], while downregulated plasma lncRNA cancer susceptibility 11 (CASC11) in patients with coronary artery disease indicated an active disease state and was closely associated with high mortality rates [7]. LncRNA SRA1 (SRA1) dysregulation in heart failure was initially observed in an expression profiling study of abnormally expressed lncRNAs in ischemic heart failure [8]. SRA1 was reported to be a cardiomyopathy risk factor and was linked with cardiac myofibroblast activation [9, 10]. Furthermore, SRA1 was observed to regulate numerous physiological and pathological processes, such as steroidogenesis, tumorigenesis, hepatic steatosis, and stem cell differentiation [11, 12, 13]. Moreover, alerted SRA1 expression was detected in other diseases, indicating its vital role in disease development and outcomes. For example, SRA1 dysregulation was found in patients with uterine leiomyoma tumors, where it showed the ability to discriminate different phenotypes [14]. The rs10463297 single-nucleotide polymorphism of SRA1 is also closely related with the susceptibility of developing polycystic ovary syndrome [15]. Lastly, SRA1 modulation changes have been observed in cardiac myofibroblasts, hypoxia-induced cardiomyocyte injury, and hepatocellular carcinoma [10, 16, 17]. Therefore, SRA1 is a potential biomarker of CHF.

This study investigated the discriminative and predictive value of SRA1 in CHF, aiming to present a novel biomarker for the early detection and prognosis prediction of CHF.

## 2. Materials and Methods

### 2.1 Patient and Controls Enrollment

This study included 93 hospitalized patients who were diagnosed with chronic heart failure from January 2020 to December 2021. The inclusion and diagnosis criteria for enrolling CHF patients were as follows:

(1) Typical chronic heart failure symptoms, including exertional dyspnea/upright respiration, nocturnal paroxysmal dyspnea, or symptoms of organ under-perfusion, such as fatigue and compensatory tachycardia, pulmonary auscultation (rales), lower limb edema, and a prominent or bulging jugular vein.

(2) Patients were primarily diagnosed with chronic heart failure according to the 2018 Guidelines for Diagnosis and Treatment of Heart Failure in China. Based on the left ventricular ejection fraction (LVEF), the phenotypes of the enrolled patients were divided into HF with reduced ejection fraction (HFrEF, LVEF ≤40%), HF with mildly reduced ejection fraction (HFmrEF, LVEF 41–49%), and HF with preserved ejection fraction (HFpEF, LVEF ≥50%). Among the HFrEF patients, the LVEF increased by ≥10% after treatment, with a post-therapy LVEF of >40% defined as HF with improved EF (HFimpEF).

(3) Chronic heart dysfunction indicated by an echocardiogram (LVEF of <40% indicating systolic dysfunction and blood flow spectrum of the mitral orifice with decreasing peak E, increasing peak A, and decreasing E/A ratio suggesting abnormal diastolic function).

(4) Patients with completed clinical records.

The exclusion criteria were:

(1) Age below 18 years.

(2) Presence of any comorbidities, including malignant tumors, renal dysfunction, severe primary diseases, and acute or chronic infections.

(3) Acute myocardial infarction within the past 1 month.

(4) Hemodynamical instability as indicated by abnormalities in the superior cava arterial blood pressure, heart rate, central venous pressure, right arterial pressure, and right ventricular pressure.

Another 62 healthy individuals that are free from any disease events were enrolled as the control group using the following inclusion criteria:

(1) No apparent liver or kidney dysfunction, and no abnormalities on physical examinations.

(2) No primary heart diseases, as confirmed by one of the following methods: echocardiography, coronary angiography, or radionuclide imaging examination.

The modified events per variable equation (N = 10 × k/p), was used to calculate the sample size, where N represented the sample size; k, the number of independent variables; and p, the loss to follow-up rate of the study participant. The study protocol was approved by the Ethics Committee of Weifang People’s Hospital (No.2019025). All procedures performed were in accordance with the 1964 Helsinki Declaration and its later amendments. Informed consent was obtained from all included participants.

### 2.2 Echocardiographic Examination

Echocardiographic evaluation was conducted using a cardiac color Doppler ultrasound scanner (Agger vascular E95 color Doppler ultrasound diagnostic instrument, GE Healthcare, Tokyo, Japan) and M5Sc-D probe (GE Healthcare, Tokyo, Japan). During the imaging process, patients were maintained in the supine position with their right arm raised above their head. The heart structure was examined near the left ventricular sternum in a long axial position. The 2D images were captured at a frame rate of 54–65 frames/s, yielding a minimum of 33 dynamic 2D and M-mode ultrasound images. Additionally, the Simpson method was applied to the images to measure the LVEF.

### 2.3 Follow-up Survey

All patients were followed up for 6 months via telephone or outpatient review using a structured interview. Event-free survival, defined as no disease development or disease-induced deaths, was evaluated among all patients. The follow-up information comprised changes in patients’ symptoms and exercise tolerance, recurrence and readmission conditions, and survival data. The diagnostic criteria for CHF recurrence mainly included pronounced chest tightness, wheezing, difficulty breathing, orthopnea, occasional expectoration of copious pink bubble sputum, and the aforementioned diagnostic criteria for CHF.

### 2.4 Sample Collection and SRA1 Detection

Venous blood samples were collected from each participant in anticoagulation tubes containing ethylene diamine teraacetic acid (EDTA). The samples were then centrifugated at 3000 rpm for 15 min to isolate the plasma, followed by chemiluminescence immunoassays to analyze the BNP levels.

The *SRA1* levels were estimated via real-time quantitative polymerase chain reaction (PCR). Initially, the plasma samples were lysed, and the total RNA was extracted with Trizol reagent (catalog# 15596026, Invitrogen, Grand Islan, NY, USA). Next, cDNA was generated using the SuperScript IV First-Strand Synthesis System (18091050; Invitrogen, Carlsbad, CA, USA). Further, the CFX96 Touch Real-time PCR detection system (lot number 1855195, Bio-Rad, Hercules, CA, USA) was employed for evaluating *SRA1* with the following primers: *SRA1* F-GCTGGGCACTGGGAATGTAA, R-CACGACCCTACAACCCTCTG; *GAPDH* F-AGAAGGCTGGGGCTCATTTG, and R-GCAGGAGGCATTGCTGATGAT. The relative expression level of *SRA1* was calculated based on the $2^{-\Delta \,\Delta \,\text{Ct}}$ method normalized to *GAPDH* mRNA.

### 2.5 Statistical Analysis

The Kolmogorow-Smirnov test was employed to evaluate the normal distribution of data (**Supplementary Table 1**). The data were expressed as mean ± standard deviation (SD). Data comparisons were conducted utilizing the Student’s *t*-test and one-way analysis of variance (ANOVA) (*p* < 0.05). SPSS26.0 software (IBM, Armonk, NY, USA) was used to perform statistical analyses, including receiver operating curve (ROC) analysis to assess the discriminating ability of SRA1 in distinguishing CHF patients from healthy individuals and the Kaplan-Meier analysis and multivariate Cox regression analyses to evaluate the role of SRA1 in CHF. Moreover, GraphPad Prism 9.0 (GraphPad Software, La Jolla, CA, USA) was utilized to analyze the correlation between SRA1 and the patients’ clinicopathological features with the Spearman correlation analysis.

## 3. Results

### 3.1 Basic Characteristics of Study Subjects

Age, body mass index (BMI), sex, hypertension, and diabetes history were matched between healthy individuals and chronic heart failure patients (*p* >0.05, Table 1). CHF patients showed significantly higher BNP levels (812.47 ± 20.68 *vs.* 46.73 ± 9.29 pg/mL), left atrial end-systolic diameter (LAD) (48 ± 13 *vs.* 28 ± 6 mm), and left ventricular end-diastolic diameter (LVDd) (66 ± 8 *vs.* 37 ± 8 mm) and lower LVEF (34 ± 8% *vs.* 64 ± 10%) than the healthy individuals (*p* < 0.001, Table 1). Furthermore, CHF patients were primarily caused by coronary heart disease (n = 46), myocardial infarction (n = 24), hypertension (n = 15), and arrhythmia (n = 8). According to the LVEF, the phenotype of the enrolled CHF patients included 71 HFrEF, 21 HFmrEF, and 1 HFpEF patient. Among the HFrEF patients, the LVEF of 29 patients increased by ≥10% post-treatment, with a post-therapy LVEF >40% defined as HFimpEF (Table 1).

**Table 1.** **Basic clinical features of study subjects**.

		Healthy individuals	Chronic heart failure patients	*p*
Age		65 ± 12	63 ± 10	0.397
BMI		24.68 ± 2.50	25.25 ± 3.14	0.235
Sex (M/F)		38/24	57/36	0.758
Hypertension (n, %)		29 (47)	48 (51)	0.555
Diabetes (n, %)		15 (24)	33 (35)	0.136
BNP (pg/mL)		46.73 ± 9.29	812.47 ± 21	<0.001
Albumin (g/L)		42.61 ± 3.29	34.89 ± 3.72	<0.001
CRP		0.77 ± 0.13	6.60 ± 1.07	<0.001
LAD (mm)		28 ± 6	48 ± 13	<0.001
LVEF (%)		64 ± 10	34 ± 8	<0.001
LVDd (mm)		37 ± 8	66 ± 8	<0.001
Etiologies				
NYHA grades				-
	I	-	0	-
	II	-	47	-
	III	-	38	-
	IV	-	8	-
Pulmonary infection (n)		-	53	-
	Overwork	-	12	-
	Others	-	28	-

All data (except for sex, hypertension, diabetes, and etiologies) were expressed as mean ± SD; sex composition was expressed as the number of males (M) and females (F); hypertension and diabetes were expressed as number (n) and percentage (%); etiologies were expressed with corresponding numbers of patients. BMI, body mass index; BNP, brain natriuretic peptide; LAD, left atrial end-systolic diameter; LVEF, left ventricular ejection fraction; LVDd, left ventricular end-diastolic diameter; SD, standard deviation; CRP, C-reactive protein; NYHA, New York Heart association.

### 3.2 Plasma SRA1 in CHF Patients

**Fig. 1.**
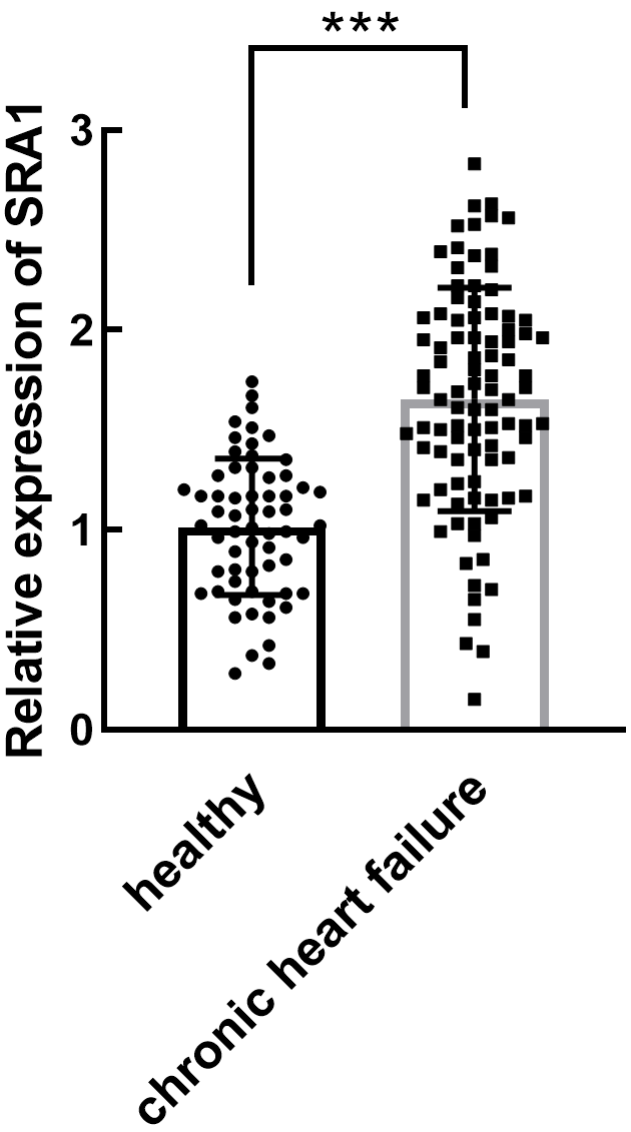
**Expression of lncRNA SRA1 in CHF patients and non-heart failure individuals**. Significant upregulation of SRA1 was observed in CHF patients. mean ± SD. ***p < 0.001. lncRNA, long non-coding RNA; SRA1, steroid receptor RNA activator 1; CHF, chronic heart failure; SD, standard deviation.

**Fig. 2.**
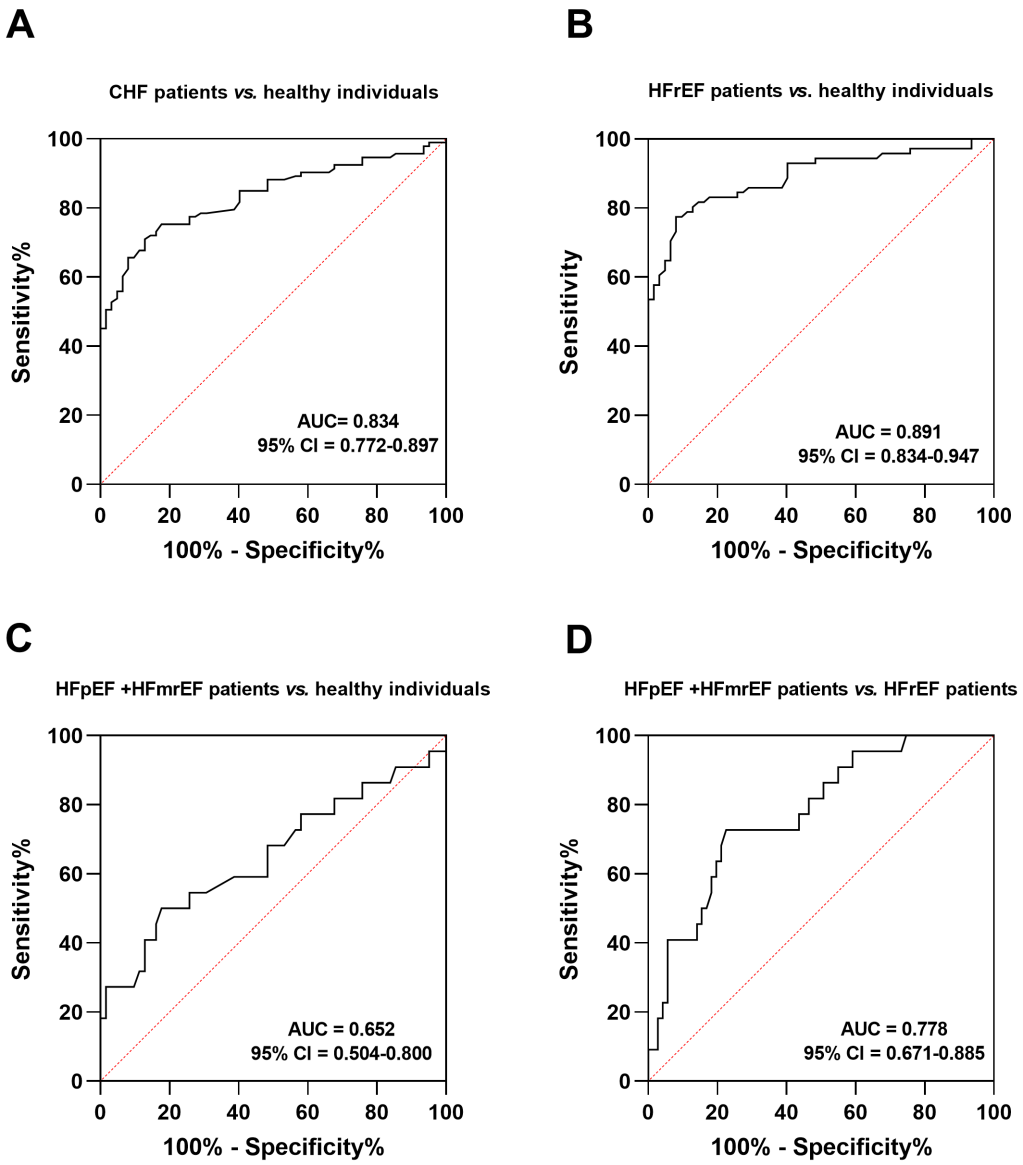
**ROC analysis to evaluate the diagnostic significance of SRA1**. (A) CHF patients *vs.* healthy individuals. (B) HFrEF patients *vs.* healthy individuals. (C) HFpEF and HFmrEF patients *vs.* healthy individuals. (D) HFpEF and HFmrEF patients *vs.* HFrEF patients. ROC, receiver operating curve; HF, heart failure; HFrEF, HF with reduced ejection fraction; HFpEF, HF with preserved ejection fraction; HFmrEF, HF with mildly reduced ejection fraction; AUC, area under curve; CI, confidence interval; SRA1, steroid receptor RNA activator 1; CHF, chronic heart failure.

CHF Patients had higher plasma SRA1 expression levels (mean = 1.65) than healthy individuals (mean = 1.01, *p* < 0.001, Fig. 1), indicating its potential role in the pathogenesis and development of CHF. Based on the mean SRA1 plasma level of CHF patients, the patients were divided into two groups a low SRA1 group (46 patients) and a high SRA1 group (47 patients). Moreover, SRA1 could discriminate CHF patients from healthy individuals, achieving a sensitivity and specificity of 0.710 and 0.871, respectively (Fig. 2A, area under curve (AUC) = 0.834). For the different subtypes of CHF patients, SRA1 also showed significant potential in discriminating HFrEF patients (AUC = 0.891, Fig. 2B) and HFpEF and HFmrEF patients (AUC = 0.652, Fig. 2C) from healthy individuals. More importantly, SRA1 could also distinguish between HFrEF patients and HFpEF and HFmrEF patients (AUC = 0.778, Fig. 2D).

**Fig. 3.**
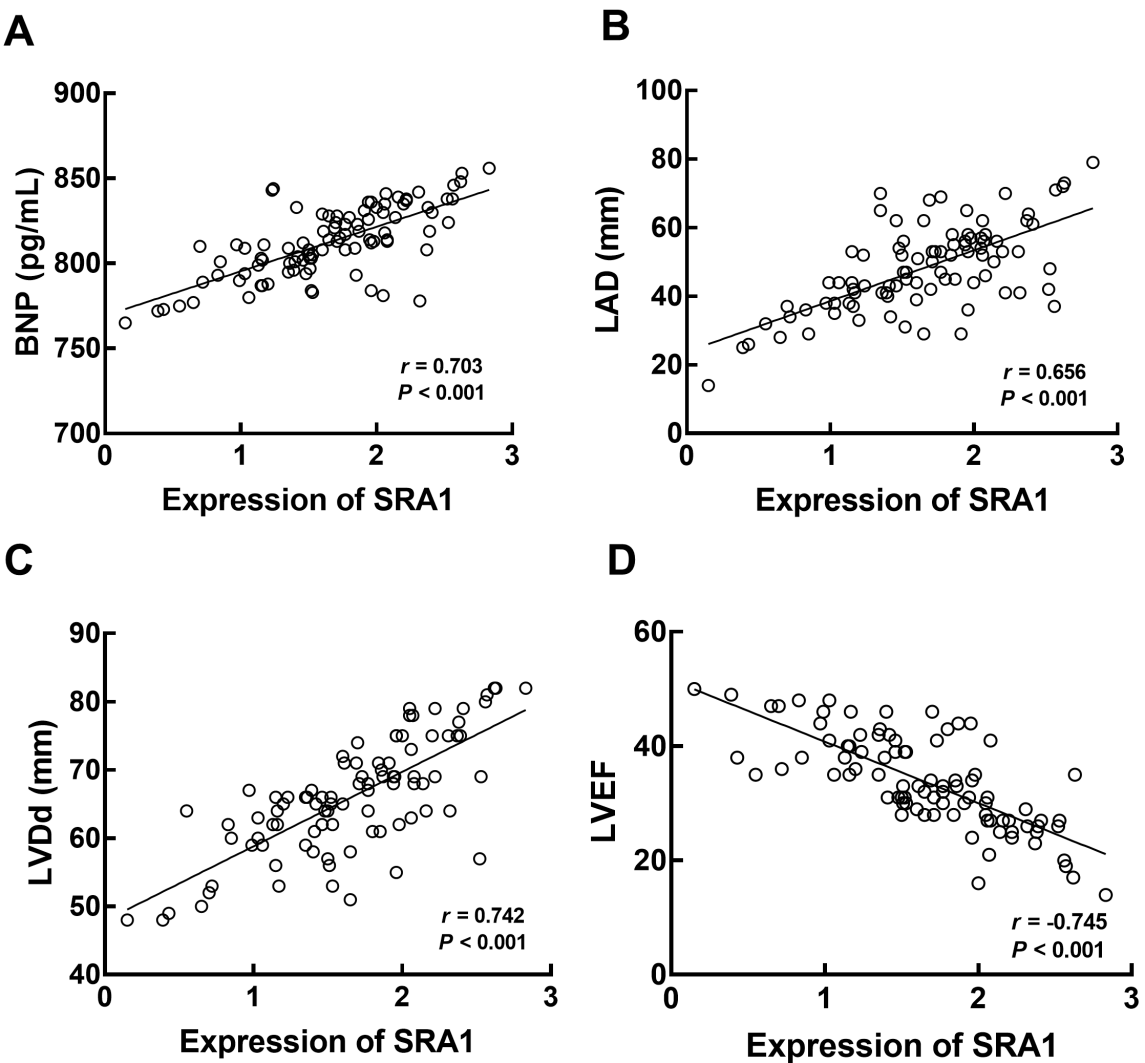
**Correlation of SRA1 with the BNP (A), LAD (B), LVDd (C), and LVEF (D) of CHF patients**. Positive correlations between SRA1 and BNP (*r* = 0.703), LAD (*r* = 0.656), LVDd (*r* = 0.742), and negative correlation with LVEF (*r* = –0.745) were observed. *p* < 0.001. SRA1, steroid receptor RNA activator 1; CHF, chronic heart failure; BNP, brain natriuretic peptide; LAD, left atrial end-systolic diameter; LVEF, left ventricular ejection fraction; LVDd, left ventricular end-diastolic diameter.

SRA1 showed significant positive correlations with BNP (*r* = 0.703, Fig. 3A), LAD (*r* = 0.656, Fig. 3B), and LVDd (*r* = 0.742, Fig. 3C), and a negative correlation with LVEF (*r* = –0.745, Fig. 3D, *p* < 0.001).

### 3.3 Prognostic Value of SRA1 in CHF Patients

**Fig. 4.**
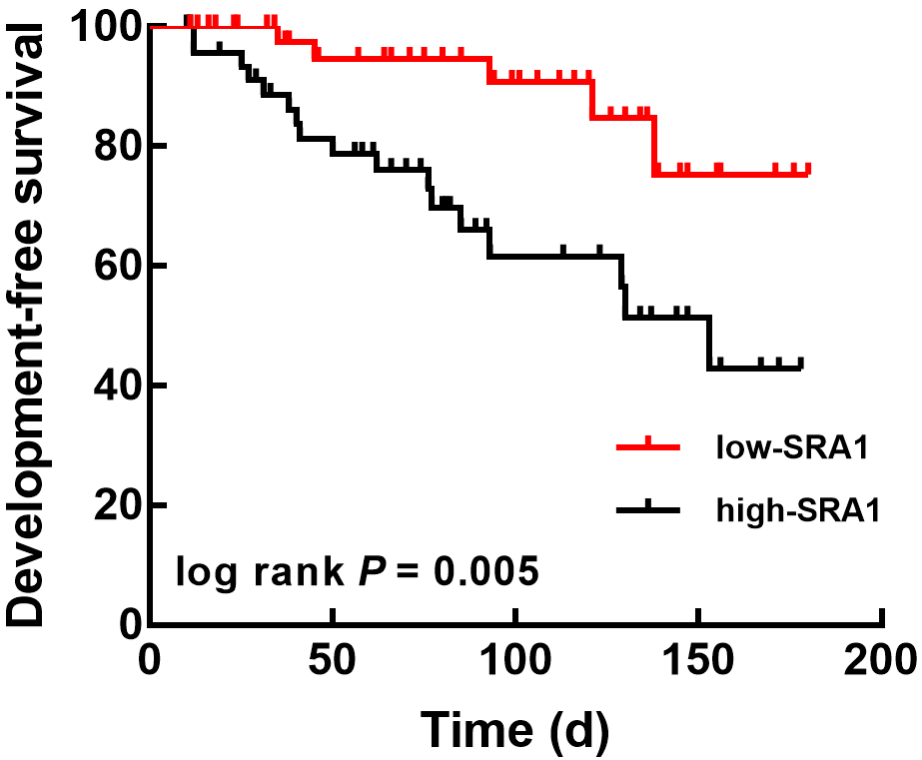
**Kaplan-Meier analysis to evaluate the association of SRA1 with the event-free survival of CHF patients based on the plasma SRA1 level**. The relatively high expression of SRA1 was significantly associated with the development-free survival of CHF patients. log-rank *p* = 0.005. Development-free survival: patients without any disease development or any causes-induced deaths. SRA1, steroid receptor RNA activator 1; CHF, chronic heart failure.

The endpoints of the follow-up survey were heart failure recurrence, heart failure-related readmission, acute myocardial infarction, severe arrhythmia, and cardiac death. There were no death cases in the low-SRA1 group, but 4 death cases were followed up in the high-SRA1 group (23.53%). Patients in the high SRA1 group showed a poorer event-free survival rate with the median survival of 153 days than those in the low SRA1 group (hazard ratio, HR = 3.313, 95% confidence interval (CI) = 1.429–7.681, log-rank *p* = 0.005, Fig. 4).

Lastly, Cox regression analysis demonstrated that plasma SRA1 level was an independent indicator of adverse outcomes in CHF patients with an HR factor of 6.020 (95% CI = 1.196, *p* = 0.029) along with LAD (HR = 5.096, 95% CI = 1.947–18.621, *p* = 0.033), LVEF (HR = 4.107, 95% CI = 1.096–15.392, *p* = 0.036), and LVDd (HR = 4.157, 95% CI = 1.095–15.780, *p* = 0.036) as additional independent predictors (Table 2).

**Table 2.** **Cox regression analysis evaluating the prognostic value of clinical features of patients**.

	HR	95% CI	*p*
lncRNA SRA1	6.020	1.196–30.296	0.029
Age	1.636	0.544–4.917	0.381
BMI	1.933	0.704–5.304	0.201
Sex	1.343	0.453–3.987	0.595
Hypertension	1.639	0.613–4.381	0.325
Diabetes	2.565	0.930–7.069	0.069
BNP	3.513	0.677–18.231	0.135
LAD	5.096	1.947–18.621	0.033
LVEF	4.107	1.096–15.392	0.036
LVDd	4.157	1.095–15.780	0.036

HR, hazard ratio; CI, confidence interval; lncRNA, long non-coding ribonucleic acid; SRA1, steroid receptor RNA activator 1; BMI, body mass index; BNP, brain natriuretic peptide; LAD, left atrial end-systolic diameter; LVEF, left ventricular ejection fraction; LVDd, left ventricular end-diastolic diameter.

## 4. Discussion

The diagnostic and prognostic value of lncRNAs in human diseases has been substantially validated, with the dysregulation of several lncRNAs being demonstrated as biomarkers for heart failure [18]. For instance, lncRNA lung cancer associated transcript 1 (LUCAT1) was identified as a biomarker for CHF that assists the diagnosis and prognosis prediction of CHF [19]. A previous study identified that a cytoplasmic lncRNA Caren antagonizes heart failure by suppressing DNA damage and promoting mitochondrial biogenesis [20]. Plasma lncRNA myosin heavy-chain-associated RNA transcripts (MHRT) has also been shown to predict survival in CHF patients implying its role as a diagnosis and prognosis biomarker [21]. In this study, it was found that compared with healthy individuals, CHF patients had upregulated plasma lncRNA SRA1 consistent with prior research results [8]. Moreover, SRA1 upregulation exhibited a significant potential in diagnosing CHF patients, demonstrating high sensitivity and specificity in differentiating CHF patients from healthy individuals. Abnormal BNP levels, LAD, LVDd, and rLVEF volume are established diagnostic markers of CHF, and these parameters correspond to other pathogenetic factors, including respiratory failure, pneumonia, and chronic kidney dysfunction [22, 23, 24, 25, 26]. Additionally, a previous study demonstrated BNP and LVEF could predict the appropriate therapy and hospitalization in HF patients receiving internal cardioverter defibrillator (ICD) and identified a novel protein, soluble growth stimulation expression gene 2 protein (ST2) as a biomarker for the therapeutic efficiency of ICD [27]. However, the detection of these indicators requires complex examination processes and professional expertise for interpreting results and lacks sensitivity and specificity to screen CHF. In contrast, measuring serum SRA1 levels is relatively simpler owing to the simple analytical methods and easily acquired samples. Therefore, serum SRA1 could be used to screen CHF onset much earlier, highlighting the potential role of serum SRA1 in enhancing the diagnostic efficiency of traditional indicators utilized in the clinic. The observed significance of SRA1 in CHF diagnosis also implies its potential in predicting therapeutic efficiency, which needs validation with expanding study subjects. In addition to the advantages of serum SRA1 in the early detection of CHF, this study observed significant correlations between SRA1 and the above indicators, which are considered critical indicators of disease progression [28, 29, 30, 31, 32]. Previously, SRA1 has been suggested to predict hepatocellular carcinoma (HCC) development due to its significant association with tumor size and serum glucose level, indicating that SRA1 may be a novel biomarker of HCC [16]. SRA1 has also been proposed as an indicator of adverse prognosis in ovarian cancer and HCC [16, 33]. A previous multiple-center research evaluated the effect of telemonitoring on the clinical outcomes of CHF patients found that telemonitoring could predict the hospitalization of CHF patients, but showed no significant effect on overall mortality and cardiac death events [34]. Although telemonitoring is a novel and reliable strategy for predicting patients’ prognosis, it is still of hysteresis. The current study results demonstrated that upregulated SRA1 was closely associated with poor event-free survival in CHF patients and may be an independent indicator of patient prognosis as well as the standard clinical prognostic indicators LAD, LVEF, and LVDd, providing a more advanced biomarker for CHF prognosis. Additionally, due to the close association of SRA1 with LVEF of CHF patients, SRA1 was also revealed to distinguish CHF patients with different subtypes, including HFrEF, HFmrEF, and HFpEF patients. Recent studies have revealed various therapeutic strategies for improving the prognosis of HF. Therefore, elevated serum SRA1 levels could serve as a biomarker for determining CHF incidence and detecting severe development of CHF.

CHF arises from a myriad of factors and involves a complex pathophysiological process. Among the various pathogenetic factors, apoptosis and necrosis of cardiomyocytes have been suggested as crucial contributors to the development of this disease [35]. Previous researchers have also investigated the role of lncRNAs in cardiomyocyte injury. For example, downregulated lncRNA Growth-arrest-associated long non-coding RNA1 (GASL1) in CHF was demonstrated to regulate cardiomyocyte apoptosis, and therefore mediate the development of CHF [36]. miR-1246 was reported to accelerate myocardial angiogenesis in CHF, exerting a significant protective effect [37]. Furthermore, SRA1 was found to alleviate the hypoxia-induced injury of cardiomyocytes, signifying its importance in treating hypoxia-induced heart failure [17]. The prognosis of CHF is associated with various factors. Hyperglycemia and diabetes-related metabolic disorders would induce inflammation and reduce LVEF, which further caused adverse prognosis of patients. Recent study identified functional molecules in diabetes could regulate cardiomyocytes and are of great potential to regulate cardiovascular disease [38, 39]. Anti-diabetes drug could also alleviate systemic inflammation and improve BNP levels, and therefore improve patients’ prognosis [40, 41]. Pleiotropic anti-remodeling effects of drugs also play critical roles in CHF. For instance, angiotensin receptor/Neprilysin inhibitors regulated the expression of inflammation-related miRNAs, and further affect the anti-remodeling effects of defibrillator [42]. However, extensive investigations on the role of SRA1 in these processes are lacking in the present study. Thus, future studies should focus on exploring the association of SRA1 level with the varied pathogenetic factors involved in CHF. This approach may help to markedly augment the therapeutic strategies for CHF. For example, the modulatory effect of SRA1 on signal transduction is a central mechanism underlying its functional role in cells. Hence, future research should be directed toward examining the downstream signaling pathway linked to the role of SRA1 in CHF. Moreover, this study is a single-center study with a relatively small sample size, which might limit the clinical findings. Future studies involving larger sample sizes are necessary to minimize systematic errors.

## 5. Conclusions

According to the above findings, the upregulation of lncRNA SRA1 can be an efficient biomarker to discriminate CHF patients and different subtypes. It can also predict patient prognosis, emphasizing that SRA1 is a promising indicator of CHF.

## Data Availability

The datasets used and/or analysed during the current study are available from the corresponding author on reasonable request.

## Abbreviations

BNP, brain natriuretic peptide; HfpEF, heart failure with preserved ejection fraction; HfrEF, heart failure with reduced ejection fraction; LAD, left atrial end-systolic diameter; lncRNAs, long non-coding RNAs; LVDd, left ventricular end-diastolic diameter; NT-proBNP, NT-prosomal BNP; SRA1, lncRNA SRA1.

## Supplementary Material

Supplementary material associated with this article can be found, in the online version, at https://doi.org/10.31083/j.rcm2505178.
