# Supplementary material for: Diagnostic and Prognostic Value of Plasma lncRNA SRA1 in Chronic Heart Failure
Source: Rev Cardiovasc Med. 2024 May 20;25(5):178. doi: 10.31083/j.rcm2505178 (PMC11267213; doi:10.31083/j.rcm2505178)
Supplement: Supplementary file 1 [file 2153-8174-25-5-178-s1.docx]

Supplementary Table 1. Kolmogorov-Smirnov test evaluating the normal distribution of data.

|  | KS distance | *P*-value |
| --- | --- | --- |
| SRA1 expression in healthy individuals | 0.071 | > 0.10 |
| SRA1 expression in CHF patients | 0.051 | > 0.10 |
| Age of CHF patients | 0.075 | > 0.10 |
| BMI of CHF patients | 0.053 | > 0.10 |
| BNP of CHF patients | 0.059 | > 0.10 |
| albumin of CHF patients | 0.066 | > 0.10 |
| CRP of CHF patients | 0.070 | > 0.10 |
| LAD of CHF patients | 0.069 | > 0.10 |
| LVDd of CHF patients | 0.075 | > 0.10 |
| LVEF of CHF patients | 0.086 | 0.086 |
| Age of healthy individuals | 0.066 | > 0.10 |
| BMI of healthy individuals | 0.081 | > 0.10 |
| BNP of healthy individuals | 0.097 | > 0.10 |
| albumin of healthy individuals | 0.076 | > 0.10 |
| CRP of healthy individuals | 0.079 | > 0.10 |
| LAD of healthy individuals | 0.081 | > 0.10 |
| LVDd of healthy individuals | 0.056 | > 0.10 |
| LVEF of healthy individuals | 0.082 | > 0.10 |
